# Intravital FRET Imaging of Tumor Cell Viability and Mitosis during Chemotherapy

**DOI:** 10.1371/journal.pone.0064029

**Published:** 2013-05-15

**Authors:** Aniek Janssen, Evelyne Beerling, René Medema, Jacco van Rheenen

**Affiliations:** 1 Division of Cell Biology, The Netherlands Cancer Institute, Amsterdam, The Netherlands; 2 Department of Medical Oncology and Cancer Genomics University Medical Center Utrecht, Utrecht, The Netherlands; 3 Cancer Genomics, Hubrecht Institute-KNAW and University Medical Center Utrecht, Utrecht, The Netherlands; The Beatson Institute for Cancer Research, United Kingdom

## Abstract

Taxanes, such as docetaxel, are microtubule-targeting chemotherapeutics that have been successfully used in the treatment of cancer. Based on data obtained from cell cultures, it is believed that taxanes induce tumor cell death by specifically perturbing mitotic progression. Here, we report on data that suggest that this generally accepted view may be too simplified. We describe a high-resolution intravital imaging method to simultaneously visualize mitotic progression and the onset of apoptosis. To directly compare *in vitro* and *in vivo* data, we have visualized the effect of docetaxel on mitotic progression in mouse and human colorectal tumor cell lines both *in vitro* and in isogenic tumors in mice. We show that docetaxel-induced apoptosis *in vitro* occurs via mitotic cell death, whereas the vast majority of tumor cells in their natural environment die independent of mitotic defects. This demonstrates that docetaxel exerts its anti-tumor effects *in vivo* through means other than mitotic perturbation. The differences between *in vitro* and *in vivo* mechanisms of action of chemotherapeutics may explain the limited response to many of the anti-mitotic agents that are currently validated in clinical trials. Our data illustrate the requirement and power of our intravital imaging technique to study and validate the mode of action of chemotherapeutic agents *in vivo*, which will be essential to understand and improve their clinical efficacy.

## Introduction

Taxanes are among the most widely used chemotherapeutics in the treatment of cancer for over a decade [1]. Taxanes, such as paclitaxel (Taxol) and its more potent, semi-synthetic analogue docetaxel (Taxotere) have been shown to bring clinical benefit in various types of cancer [2]–[4]. However, only half of the cancer patients eventually respond to docetaxel treatment [3], indicating that a better understanding of the specific effects of docetaxel in tumors could help design new combination therapies and improve its efficacy.

Taxanes stabilize microtubules by binding to the beta-tubulin subunit of tubulin polymers [5]–[8]. The clinical efficacy of taxanes has mainly been ascribed to the potent inhibitory effects they have on (tumor) cell proliferation *in vitro* by delaying mitotic progression. Although variation exists in the exact timing of cell death, most tumor cell lines treated with high doses of taxanes form abnormal mitotic spindles, resulting in prolonged mitosis and eventually cell death [9]–[12]. Cell death occurs either in mitosis, which is termed mitotic cell death, or in interphase following exit from mitosis in a tetraploid state [10], [12]. Low doses of paclitaxel also affect mitotic spindle formation and induce cell death, but do not induce a severe delay in mitotic timing [13]–[15]. These low doses of paclitaxel rather induce aneuploidy (an abnormal chromosome number) in the respective daughter cells which eventually causes cell death [13].

Although various taxane concentrations induce different mitotic perturbations, a clear correlation exists *in vitro* between abnormal mitotic progression and cell death upon taxane treatment. However, data from mice and human patients challenge this idea [3], [16]–[19]. Immunohistological analysis of both mouse and human tumor tissues only revealed small increases in mitotic index (percentage of mitotic cells) following paclitaxel treatment [17]–[19]. In addition, the minor effect of paclitaxel treatment on mitotic index did not seem to correlate with tumor regression [18], [19]. However, a comprehensive comparison between *in vitro* and *in vivo* data in the same tumor model is lacking, and therefore it cannot be excluded that this discrepancy is explained by the use of different cell types. Moreover, even if mitotic perturbations would consistently precede the onset of apoptosis induced by taxanes, it would be impossible to confirm this using immunohistochemistry on fixed tissues. These techniques analyze large, fixed populations of cells and lack crucial information of the history of the group of cells undergoing mitosis and apoptosis at the time of measurement.

To overcome these technical limitations, several techniques have been developed to visualize the behavior of cells in living animals, a technique often referred to as intravital imaging [20], [21]. Using intravital imaging techniques, changes in cell behavior can be visualized during chemotherapy. For example, intravital imaging of tumor cells growing in dorsal skin fold chambers in paclitaxel-treated mice revealed that only a small percentage of tumor cells went through an aberrant mitosis [16]. Nevertheless, it is difficult to link these observations to the induction of apoptosis, since this can only be recognized when cells show the typical late apoptotic morphological changes, such as chromosome condensation and cell fragmentation. This limitation prevents the ability to monitor mitotic progression and the onset of apoptosis in the same cells before and after treatment.

Here, we report the development of high-resolution intravital imaging methods that enable the tracing of photo-marked tumor cells before and during docetaxel treatment in subsequent imaging sessions, and enable the simultaneous visualization of mitosis and the induction of apoptosis before the typical morphological apoptotic changes occur. In our assays we use docetaxel, since this drug is more potent than paclitaxel in inhibiting mitotic progression in tissue culture and is effective in killing paclitaxel-resistant tumor cells [2]. Our comparative study of *in vitro* and *in vivo* imaging data indicate that docetaxel, in contrast to its effects in cell culture, induces apoptosis *in vivo* independent of mitotic aberrations in the vast majority of cells. These data suggest that the therapeutic potency of taxanes in anti-cancer treatment could be attributed to other, mitosis-independent, detrimental effects on tumor cell viability.

## Results

### Docetaxel treatment induces cell death both *in vitro* and *in vivo*

**Figure 1:**
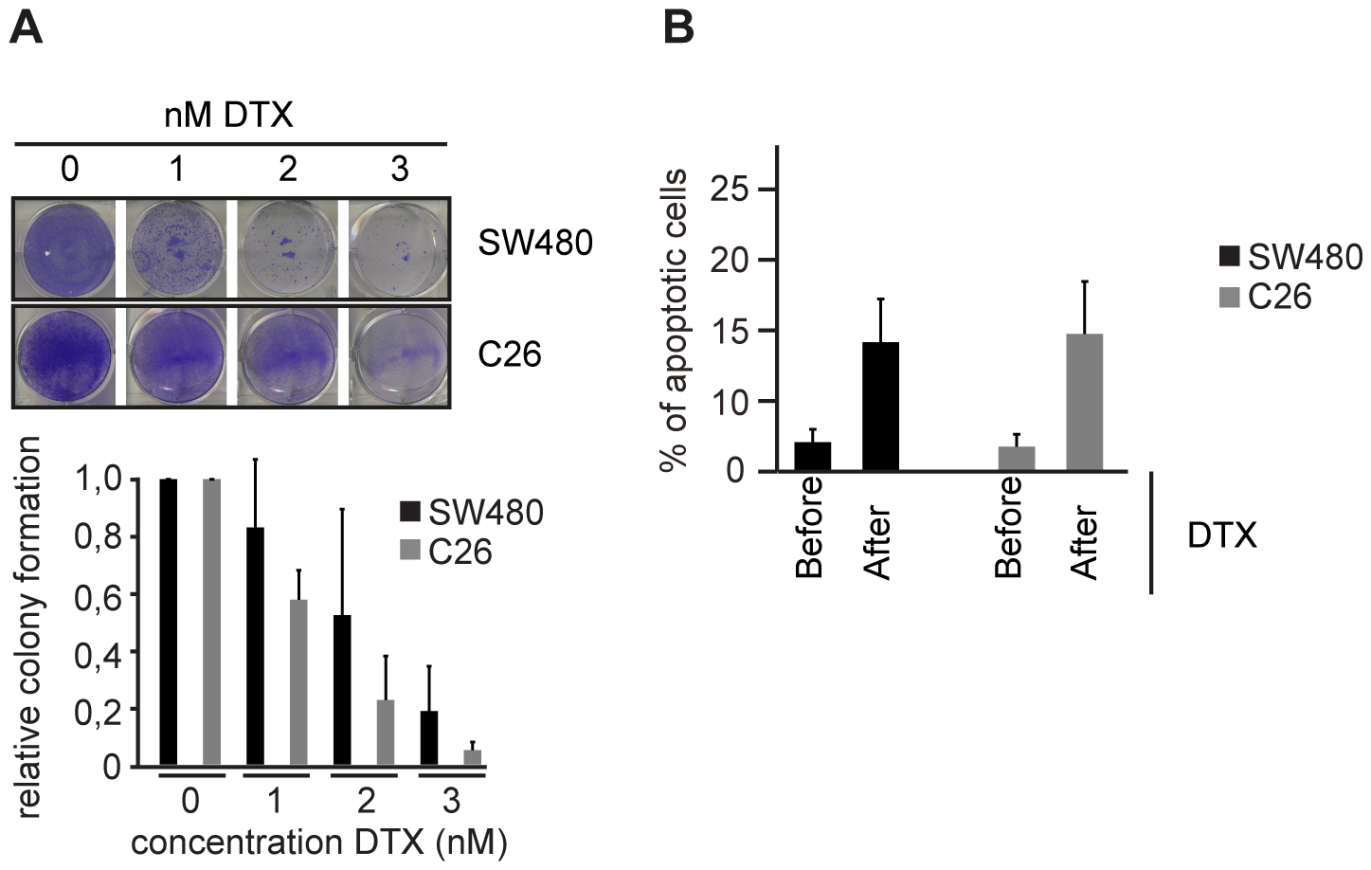
Docetaxel kills tumor cells *in vitro* and *in vivo*. A, colony formation assay of indicated cell lines treated with increasing doses of docetaxel (DTX). Colony formation capacity was determined relative to untreated controls 6 days after a single addition of docetaxel. B, quantification of the number of apoptotic cells before or 2–4 days after a single intravenous injection of 25 mg/kg docetaxel. Average of 3 independent experiments per cell line + SEM is indicated. Vehicle (PBS)-treatment is shown in Figure S1C.

For our *in vitro* and *in vivo* studies, we chose to use two colorectal tumor cell lines that can be studied *in vitro* and grow tumors upon injection in mice. We used the C26 and SW480 cell lines as a mouse isograft and human xenograft colorectal tumor model respectively. To confirm that both cell lines are sensitive to treatment with the semi-synthetic taxane docetaxel *in vitro*, we determined cell viability after several days of treatment with increasing doses of docetaxel. Colony formation capacity was clearly affected in both cell lines at a dose of 2–3 nM docetaxel, indeed showing the potency of this chemotherapeutic to kill tumor cells in tissue culture (Figure 1A).

In order to confirm the docetaxel effect *in vivo*, we subcutaneously injected C26 and SW480 cells in BalbC and immune-compromised SCID mice respectively and allowed the cells to form a tumor within 2–6 weeks. Once tumors were detectable by palpation, we treated animals once with the maximum tolerated dose of docetaxel (25 mg/kg) (Figure S1A) and visualized the number of apoptotic cells (defragmented cells) by intravital imaging (Figure S1B). In line with our *in vitro* data, we observed a substantial increase in the percentage of apoptotic cells in both tumor models (Figure 1B), which was not present in vehicle (PBS)-treated tumors (Figure S1B, C). From these data we conclude that our tumors models are sensitive to docetaxel both *in vitro* and *in vivo*.

### Simultaneously imaging mitotic progression and the onset of apoptosis

**Figure 2:**
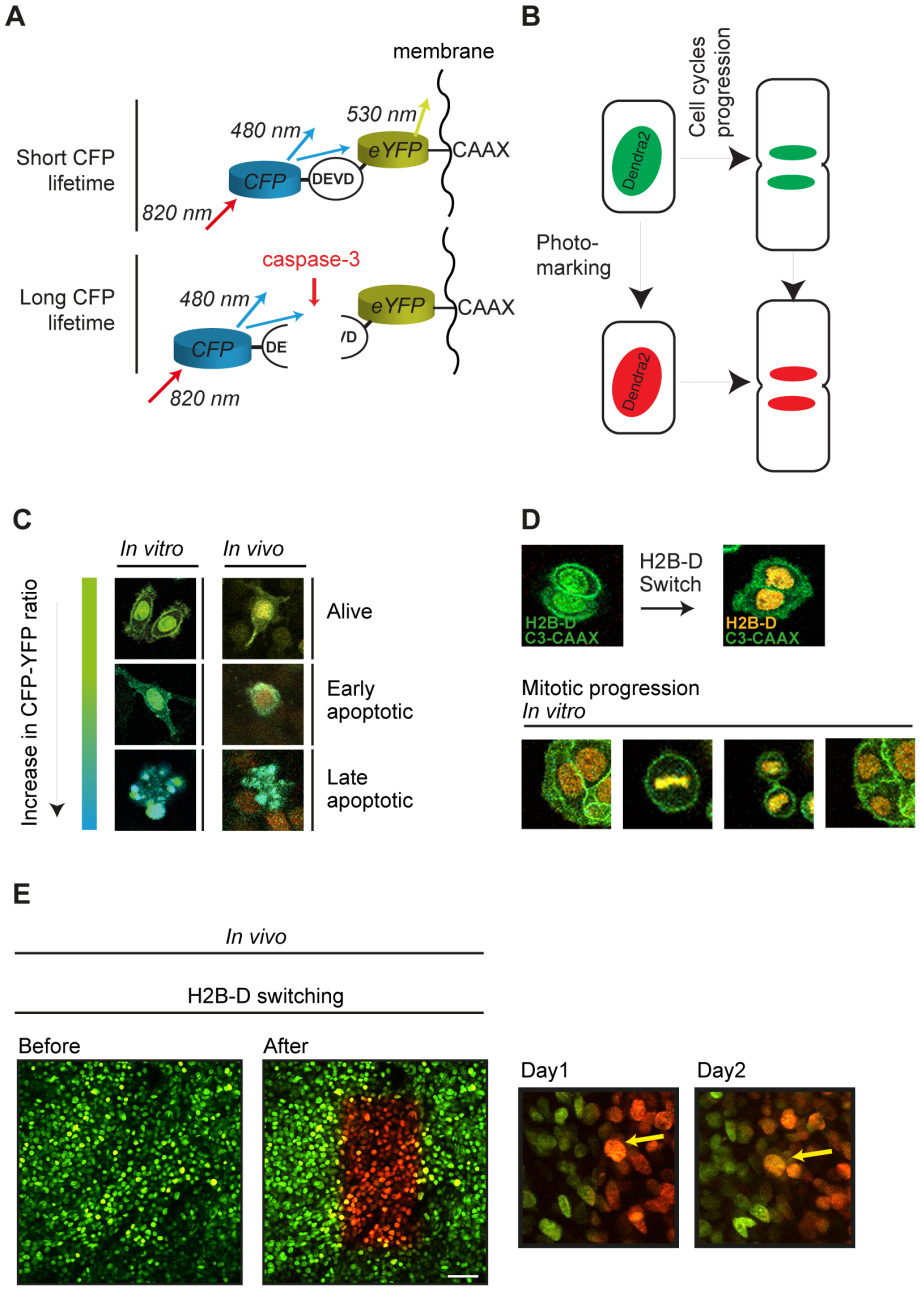
Simultaneous imaging of mitosis and the induction of apoptosis. A, schematic representation of the caspase-3 FRET probe that reports the early induction of apoptosis. In the absence of caspase-3 activity, excited CFP molecules will transfer their energy to YFP, resulting in short CFP fluorescence lifetime and YFP emission. A rise in caspase-3 activity, indicative of apoptosis induction, results in the cleavage of the DEVD motif in between the CFP and YFP moieties. This results in loss of FRET, an increase in CFP-YFP ratio and an increased CFP fluorescence lifetime. B, schematic representation of photo-marking of H2B-Dendra2 (H2B-D) cells. Switching of H2B-D from green to red enables the tracking of single cells and visualization of mitotic progression. C, color scheme and representative cells (alive, early apoptotic, late apoptotic) *in vitro* and *in vivo* showing CFP-YFP ratio changes. D, *in vitro* cells representative of H2B-D switching and mitotic progression. E, left: Representative image of H2B-D photo-switching of SW480 tumor cells *in vivo*. Scale bar represents 50 µm. Right: Stills of individual SW480 cell *in vivo* tracked at consecutive days.

To unequivocally prove that docetaxel-induced apoptosis is mediated through defects in mitotic progression, both processes should be visualized simultaneously in the same cells *in vitro* and *in vivo* before and after docetaxel treatment. Here, we have developed an imaging method to achieve this (Figure 2A and B).

Activation of the protease caspase-3 is crucial for apoptosis induction and catalyzes the cleavage of several key cellular proteins. Caspase-3 activation precedes chromosome condensation and cell fragmentation, the typical morphological changes associated with apoptotic cell death [22]. We and others have recently used a caspase-3 Fluorescent Resonance Energy Transfer (FRET) probe to visualize the onset of apoptosis *in vitro* and *in vivo* [20], [23]–[25]. The caspase-3 FRET sensor consists of a CFP and YFP moiety separated by the caspase-3 DEVD cleavage motif and is targeted to the plasma membrane by a C-terminal CAAX sequence [26] (Figure 2A and C). Under normal conditions, caspase-3 is inactive and CFP and YFP are in close proximity, so that CFP can transfer its energy to YFP. As a result of this energy transfer, CFP fluorescence will drop and YFP fluorescence will increase, leading to a low CFP to YFP ratio (referred to as CFP-YFP ratio). Caspase-3-dependent cleavage of the DEVD motif will perturb energy transfer from CFP to YFP and result in an increased CFP-YFP ratio. We created both C26 and SW480 cell lines stably expressing this apoptosis sensor and, as expected, we were able to visualize apoptosis induction both *in vitro* and *in vivo* using this caspase-3 FRET sensor (Figure 2C).

To follow both caspase-3 activation and mitotic progression before and after docetaxel treatment, we, in addition to the caspase-3 biosensor, also stably introduced Histone 2B tagged to photo-switchable Dendra2 [27] (H2B-D) in C26 and SW480 cell lines (Figure 2B and D). Mitosis can be identified when chromosomes and therefore H2B-Dendra condense. Cells without condensed H2B-Dendra were scored as interphase. In prior studies, we have used Dendra2 to specifically photo-mark and trace individual cells [28]–[30]. By switching the color of H2B-Dendra2 (H2B-D) from green to red using a violet laser [27] (Figure 2B and D), we were able to mark cells and track them both *in vitro* and *in vivo* over multiple imaging sessions (Figure 2D and E). Photo-switching of Dendra2 did not affect caspase-3 FRET measurements (Figure S2A) and more importantly, FRET levels did not change during mitotic progression, excluding possible effects of the mitotic state on FRET efficiency (Figure S2B).

To determine the apoptotic CFP-YFP ratio, the ratio at which caspase-3 activity and early apoptosis are induced, we determined the relative CFP-YFP ratio (see Materials and Methods) in cells positively and negatively stained for cleaved caspase-3 (activated form). On average, the CFP-YFP ratio was 1.3 times higher in the cleaved caspase-3-postive when compared to negative cells (Figure S2C). From here on, cells with a relative CFP-YFP ratio higher than 1.3 will be referred to as cells with an apoptotic CFP-YFP ratio.

These data show that by imaging the caspase-3 FRET sensor and H2B-Dendra2 simultaneously, we are able to visualize mitotic progression and the onset of apoptosis (1.3 times increased CFP-YFP ratio) in the same cells both *in vitro* and *in vivo*.

### Docetaxel treatment induces caspase-3 activation both *in vitro* and *in vivo*

**Figure 3:**
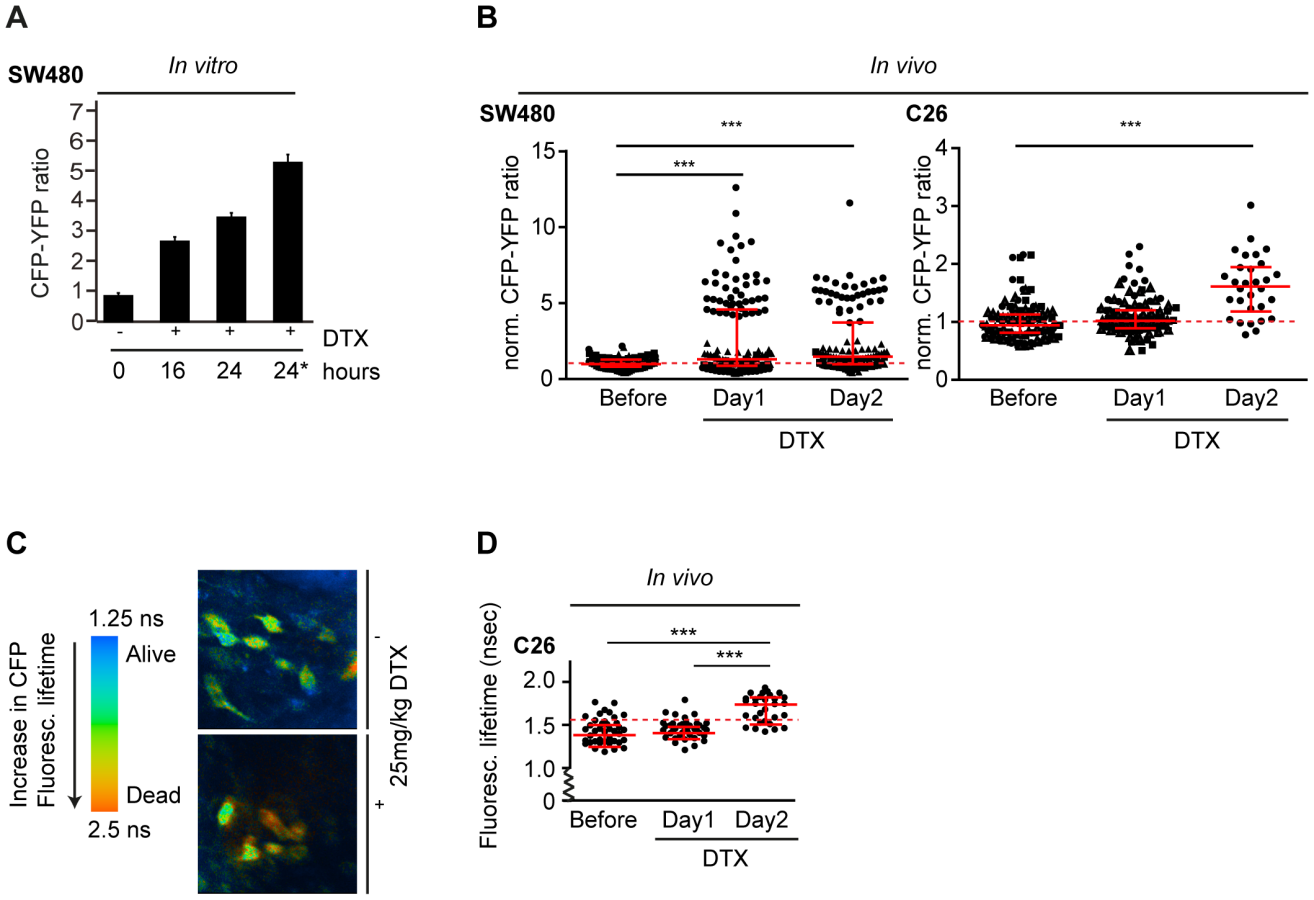
Docetaxel increases caspase-3 activity *in vitro* and *in vivo*. A, quantification of the CFP-YFP ratio of SW480 cells *in vitro* under indicated conditions (1 µm docetaxel). The 24* indicates that only apoptotic cells were analyzed, in all other cases (0, 16, and 24) mitotic cells were quantified. Apoptotic and mitotic cells were determined according to morphology. Apoptotic: membrane blebbing, fragmented DNA. Mitotic: condensed DNA, rounded membrane morphology. 10 cells were quantified per condition. Average + SEM is shown. B, quantification of the CFP-YFP ratio upon intravital imaging of tumor cells stably expressing caspase-3 FRET sensor. The CFP-YFP ratios (normalized to average CFP-YFP value before treatment) of individual cells *in vivo* of the same photoswitched tumor fields were plotted against the indicated times after a single intravenous injection of 25 mg/kg docetaxel. Red dotted line represents the average normalized CFP-YFP ratio of vehicle (PBS)-treated mice as shown in Figure S3A. Results of 3 independent experiments (visualized by different symbols for different mice) per cell line are shown. One symbol represents one cell. Line indicates median + IQR. ***: significant (Mann Whitney U test, p<0.008). C, left: Color scheme of CFP fluorescence lifetime analysis as explained in Figure 2A and C. Short lifetimes (blue) indicate high FRET levels, long lifetimes (red) indicate low FRET levels. Right: Representative FLIM images of a C26 tumor showing a group of tumor cells before (−) (upper panel) and 20 hours after (+) (lower panel) 25 mg/kg docetaxel treatment. D, the fluorescent lifetime of C26 cells *in vivo* plotted at indicated time points after docetaxel treatment. The red dotted line represents the average lifetime of vehicle (PBS)-treated mice as shown in Figure S3B. One representative experiment is shown of two independent experiments. One dot represents one cell. ***: significant (Mann Whitney U test, p<0.0001). Line indicates median + IQR.

Next, we determined the onset of apoptosis in our cell lines after docetaxel administration *in vitro*. The CFP-YFP ratio clearly increased in mitotic cells after prolonged docetaxel treatment (Figure 3A). Importantly, cells that were morphologically apoptotic (disintegrated) showed an even higher CFP-YFP ratio (5.6) when compared to the rest of the mitotic cells treated with docetaxel (3.7). This indicates that we can observe the docetaxel-induced increase in caspase-3 activity well before cells undergo the typical morphological changes associated with apoptosis (Figure 2C), which is in line with a recently proposed model in which caspase-3 activity slowly increases during a mitotic delay imposed by treatments with anti-mitotic drugs [10].

To evaluate the effects of docetaxel *in vivo*, H2B-D-expressing cells in the tumor were photo-marked and CFP-YFP ratios of single cells were subsequently determined before, 20 and 48 hours after a single intravenous docetaxel administration (25 mg/kg) (Figure 3B). This analysis revealed a significant increase in the CFP-YFP ratios of both C26 and SW480 tumor cells after docetaxel treatment when compared to pre-treated conditions (Figure 3B) and vehicle (PBS)-control (Figure S3A), indicating there is an increase in caspase-3 activity. Before docetaxel treatment, SW480 cells displayed a normalized CFP-YFP ratio of 1 on average, which increased to 2.26 and 2.34 after 20 and 48 hours of docetaxel treatment respectively. In line with these data, C26 cells also showed a significant increase in CFP-YFP ratio following docetaxel treatment (a ratio of 1.0 before and 1.09 and 1.61 after 20 and 48 hours of docetaxel treatment respectively) (Figure 3B).

To confirm our FRET results, we also performed fluorescence lifetime imaging microscopy (FLIM) (Figure 3C). Caspase-3 activation results in loss of FRET due to cleavage of the DEVD motif (Figure 2A) and therefore should cause an increase in the CFP fluorescence lifetime [31]. In line with our CFP-YFP ratio measurements, FLIM analysis of C26 cells after 25 mg/kg docetaxel treatment revealed a significant increase in CFP lifetime from, on average, 1.4 ns before docetaxel treatment to 1.7 ns at two days after docetaxel treatment (Figure 3C and D). The observed increase in caspase-3 activity was not caused by insertion of the imaging window or by light exposure during imaging, since FLIM analysis of tumor sections from within the tumor (not reached by either the window or the laser beam) showed that FRET was even more frequently lost in these areas (Figure S3B). In addition, *in vivo* FLIM analysis showed that CFP fluorescence lifetime was not increased upon vehicle (PBS) treatment (Figure S3C), confirming that both imaging and vehicle (PBS) treatment alone do not induce caspase-3 activity.

Together, these data show that caspase-3 activity, and therefore the onset of apoptosis, is induced upon treatment with docetaxel both *in vitro* and *in vivo*.

### Docetaxel-induced apoptosis depends on the mitotic status of tumor cells *in vitro* but not *in vivo*

**Figure 4:**
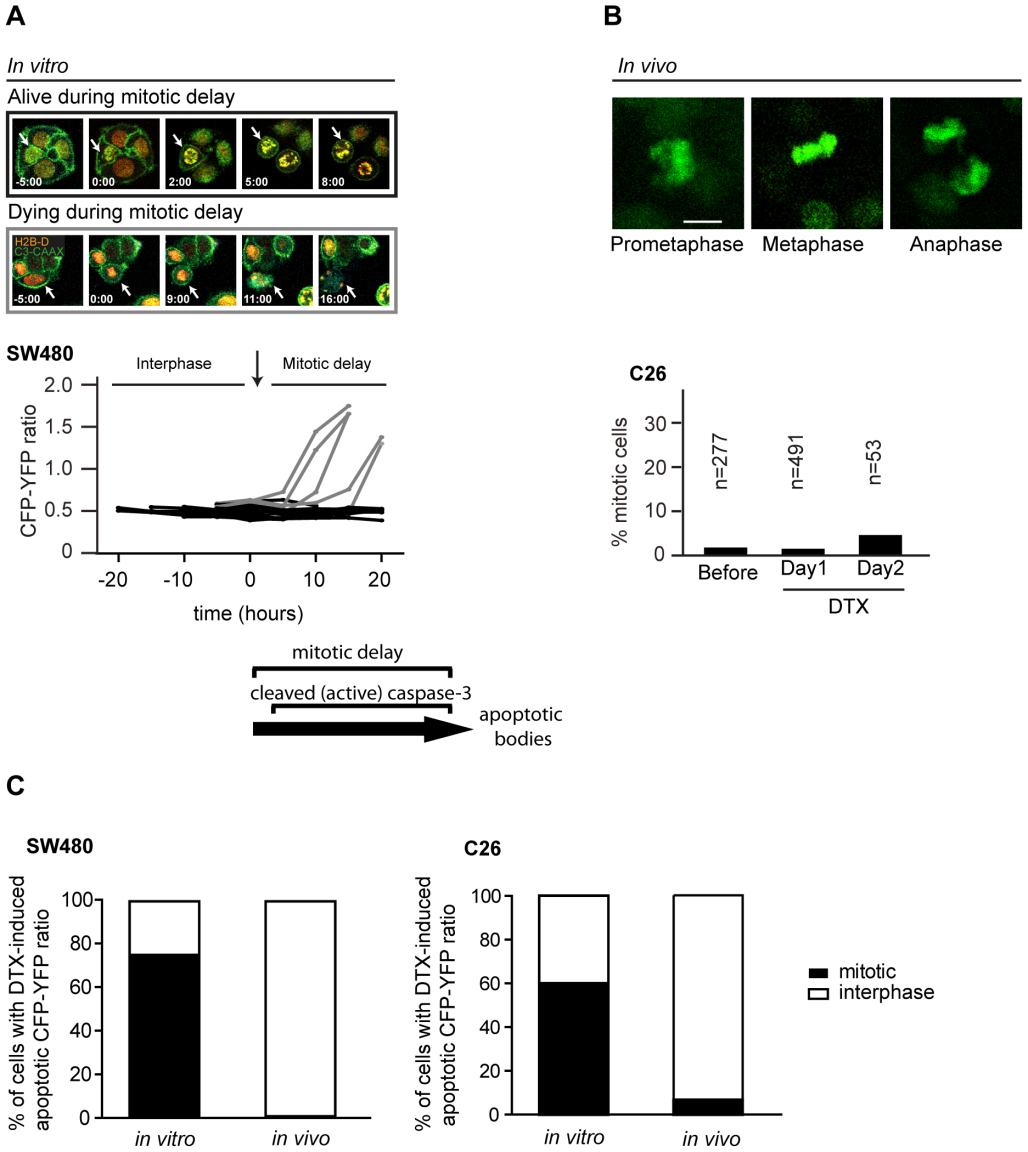
Docetaxel induces apoptosis *in vivo* independent of a mitotic delay. A, representative stills of SW480 cells *in vitro* entering mitosis in the presence of 1 µM docetaxel resulting in a mitotic delay (top) and eventually apoptosis (bottom). Bottom graph shows the CFP-YFP ratios of treated SW480 cells over time. Cells in which the CFP-YFP ratio reaches the apoptotic CFP-YFP ratio (>1.3 times increase) are indicated with gray lines and all other cells with black lines. B, left: Images of a mitotic C26 cell *in vivo* on day 2 after 25 mg/kg docetaxel treatment. Right: Quantification of the number of mitotic cells found at indicated time points (before, 20 h (day1) or 48 h (day2) after a single administration of 25 mg/kg docetaxel) in C26 tumors. Mitotic cells were determined according to nuclear morphology. C, quantification of the cell cycle state (mitotic or interphase) of cells with a docetaxel-induced apoptotic CFP-YFP ratio. The calculation of apoptotic CFP-YFP ratios (1.3 times increase) was performed as described in the Materials and Methods section. Data are plotted for both *in vitro* and *in vivo* measurements (n>20 cells per condition).

Next, we followed the effect of docetaxel treatment on mitotic progression using time-lapse imaging of both C26 and SW480 cells expressing H2B-D and the caspase-3 FRET probe (Figure 4). SW480 cells have a robust mitotic checkpoint and delay mitosis upon treatment with spindle poisons for at least 18 hours [32]. Indeed we find that docetaxel treatment *in vitro* resulted in a mitotic delay of ∼10–20 hours (Figure 4A, S4A). Of those mitotic cells, 22% showed an increase in CFP-YFP ratio (gray lines in Figure 4A) and clear apoptotic morphology during this mitotic delay (Figure 4A). The increase in CFP-YFP ratio was due to an increase in caspase-3 activity, since treatment with a pan-caspase inhibitor (z-VAD-fmk) abolished the docetaxel-induced increase in the number of cells that have an apoptotic CFP-YFP ratio (Figure S4B). Treatment of interphase cells with docetaxel *in vitro* did not affect CFP-YFP measurements (Figure S4C), which indicates that the increase in CFP-YFP ratio is specific for mitotically delayed cells. In line with previous data [9], [10], simultaneous imaging of apoptosis onset and mitotic progression shows that *in vitro* docetaxel treatment kills mitotic, but not interphase cells.

To assess the mitotic response to docetaxel *in vivo,* we analyzed the number of mitotic cells following docetaxel treatment in both C26 and SW480 tumors using intravital imaging (Figure 4B). Strikingly, we have never observed any mitotic cells in SW480 cells during the 3 hour intravital imaging sessions (data not shown). For C26 cells, only a small percentage of mitotic cells were observed in C26 tumors both before and 20 hours after docetaxel treatment (1,4% and 1,2% respectively), which slightly increased at forty-eight hours after docetaxel treatment to 3.8% (Figure 4B). By contrast, analysis of C26 tumor slides stained for the mitotic marker phospho-Histone H3 revealed that the number of cells with mitotic figures is not increased in docetaxel-treated tumors when compared to vehicle (PBS)-treated tumors (Figure S5A and B). Thus, the small increase of mitotic C26 cells *in vivo* upon docetaxel is not significant or very small.

The data above shows that docetaxel treatment *in vivo* does not lead to a clear increase in the number of mitotic cells, which suggest that for most cells, apoptosis is not mediated through a delay in mitosis. Nevertheless, if docetaxel-induced apoptosis is mediated through mitotic delay, cells should simultaneously show a continuous apoptotic CFP-YFP ratio and stay in mitotis/tetraploid state until they fragment. To test this, we analyzed the ratio of mitosis and interphase only in cells in which apoptosis (apoptotic CFP-YFP ratio) is induced by docetaxel. This analysis showed *in vivo* that less than 7% of all SW480 and C26 cells in which apoptosis is induced were in mitosis (Figure 4C). Since cells with mitotic delay should stay in mitosis until they fragment, the latter data shows that of all docetaxel-induced apoptosis (apoptotic CFP-YFP ratio), at most 7% is induced by mitotic delay and at least 93% by mitotic-independent mechanisms. In contrast, the percentage of mitotic cells of all apoptotic SW480 and C26 cells *in vitro* was 75% and 60% respectively (Figure 4C). The latter data suggest, contrary to the *in vivo* situation, that the contribution of a delayed mitotic progression to docetaxel-induced apoptosis *in vitro* is very large (Figure 4C).

### Potential mechanisms that explain the lack of induction of apoptotic delay by docetaxel

**Figure 5:**
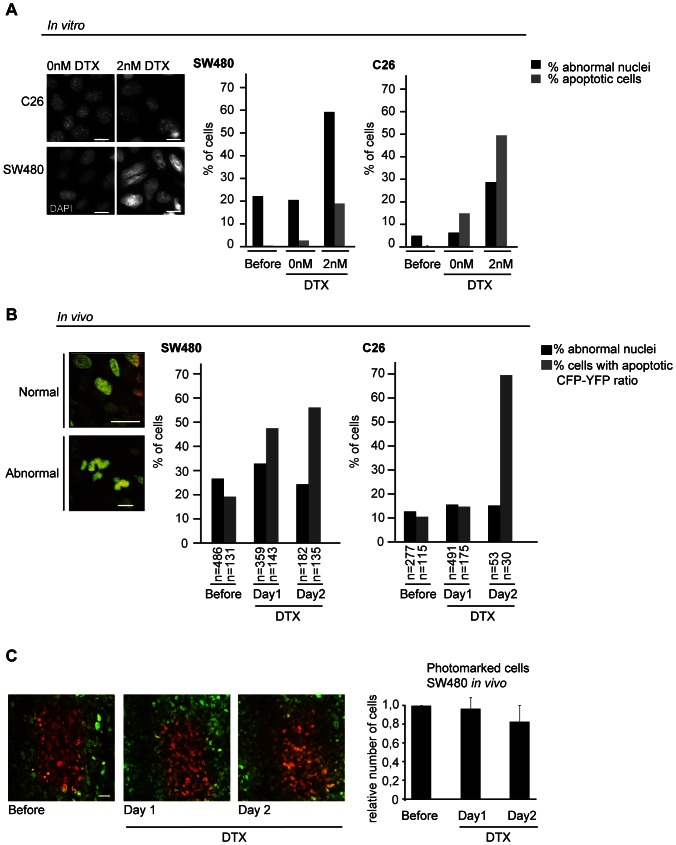
Docetaxel induces apoptosis *in vivo* independent of mitotic defects. A, left: Representative images of nuclei (DAPI) of C26 and SW480 cells *in vitro* after 1 day treatment with or without a low dose of docetaxel (2 nM). Scale bars represent 10 µm. Right: The number of C26 or SW480 cells with abnormal nuclei *in vitro* after indicated conditions. B, left: Representative images of SW480 cells *in vivo* with normal or abnormal nuclear morphology visualized using H2B-D. Scale bars represent 10 µm. Right: The number of cells with abnormal nuclei (black, see Figure S6A for representative images) and number cells with an apoptotic CFP-YFP ratio (>1.3 times increase) (gray) in SW480 and C26 tumors plotted at indicated time points (before, one day or two day after single intravenous administration of 25 mg/kg docetaxel). The n indicates number of cells analyzed. C, left: Representative images of photo-marked SW480 cells *in vivo* switched before docetaxel treatment and imaged on day 1 and 2 after single treatment. Scale bar represents 50 µm. Right: Quantification of the number of photo-marked (red) H2B-D cells in SW480 tumors *in vivo*. Relative number compared to day 0 (before treatment) is indicated. Average + SD is shown of 3 independent positions.

Two possible explanations for the absence of a clear increase in the number of mitotic cells *in vivo* are either that only low docetaxel concentrations are able to reach the tumor or that we miss mitotically delayed cells due to the relatively short imaging sessions (3 hours). Additionally, docetaxel could have a relatively short half-life *in vivo*, leading to different effects *in vivo* compared to the *in vitro* situation. To address these hypotheses, we analyzed the number of cells with an abnormal nucleus *in vitro* and *in vivo*, which is indicative of earlier mitotic defects (Figure 5A,B). Both mitotic cells treated with low doses of taxanes [13] and cells that slip out of a taxane-induced prolonged mitosis [10] undergo chromosome segregation errors, which lead to abnormal nuclei formation upon mitotic exit. Indeed, we find that low doses of docetaxel treatment *in vitro* induced the formation of abnormal nuclei and apoptosis in both C26 and SW480 cells (Figure 5A, S4A). However, *in vivo* analysis did not reveal striking differences before or after docetaxel treatment (Figure 5B, S6A). C26 tumors showed a slight increase in cells with abnormal nuclei after docetaxel treatment (12,6% before and 15,5% and 15,1% at 20 and 48 hours after docetaxel treatment respectively), but this increase did not correlate with the substantial increase in the percentage of apoptotic cells observed in the same fields (10%,14% and 70% before, 20 and 48 hours after docetaxel treatment respectively) (Figure 5B). The basal number of SW480 cells with abnormal nuclei was higher than that of C26 cells (Figure 5A,B). However, SW480 cells are intrinsically chromosome unstable, which means that they frequently lose and gain whole chromosomes during cell division, already in the absence of any treatment [33], [34]. Importantly, the number of nuclei *in vivo* with an aberrant morphology (Figure 5B) did not increase over time upon docetaxel treatment (34% and 25% of cells had an abnormal nucleus 20 and 48 hours after treatment respectively, compared to 27% before treatment). This indicates that these cells did not undergo chromosome segregation errors upon docetaxel treatment *in vivo*, whereas the percentage of apoptotic cells increased substantially in the same tumor fields (20%, 49% and 58% before, 20 and 48 hours after docetaxel treatment respectively) (Figure 5B, and see Figure S6B for the vehicle (PBS)-control). We also find that the number of photo-marked SW480 cells *in vivo* did not increase over time in docetaxel-treated tumors (Figure 5C), which provides additional proof that these cells have not divided in between the different imaging sessions. To address the hypothesis that the differences between our *in vitro* and *in vivo* data may be due to a relatively short half-life of docetaxel *in vivo*, we performed an *in vitro* docetaxel washout experiment (Figure S6C) to mimic a short exposure to docetaxel. This experiment showed that a short exposure to docetaxel still results in mitotic defects *in vitro*. Both SW480 and C26 cells that entered mitosis after docetaxel washout remained in mitosis for several hours (Figure S6C). These data indicate that docetaxel cannot be properly washed out *in vitro* and therefore most likely does not have a short half-life in tumor cells *in vivo* either. In addition, we observed an increase in mitotic cells with chromosome alignment defects *in vivo* upon docetaxel treatment, indicating that the drug did enter the tumors sufficiently long to affect mitotic progression in these cells (Figure S5C). The differences in the mode of apoptosis induction between *in vivo* and *in vitro* can therefore not be explained by a short half-life of docetaxel.

Taken together, these data show that the onset of docetaxel-induced apoptosis correlates with the number of mitotic defects *in vitro*, but not *in vivo*. From this we conclude that docetaxel, in contrast to its effect *in vitro*, can induce apoptosis *in vivo* independent of its effect on mitotic progression.

## Discussion

Although taxanes have been used in the clinic already for over a decade, a discrepancy between *in vitro* and *in vivo* data exists on this drug's therapeutic action. In this report, we have used two colorectal tumor models in which we analyze the effect of docetaxel on both mitotic progression and apoptosis induction in *in vitro* and *in vivo* settings. For this, we have developed intravital imaging techniques that enabled us to track cells in multiple imaging sessions, and simultaneously determine mitotic progression and the induction of apoptosis. For the latter one, we used a caspase-3 FRET probe that reports the initiation, not completion, of an apoptotic program. Although induction of caspase-3 has been reported to lead to cell death [22], we cannot exclude that a fraction of cells showing an apoptotic CFP-YFP ratio will not progress to death. Taken this caution into account, our method enabled us to detect the onset of apoptosis before a cell disintegrates and measure the mitotitc progression of cells. Our comparison shows that taxanes, as shown before [9]–[12], affect mitotic progression *in vitro*, but our *in vivo* data indicate that other deleterious effects are more likely to be causative in tumor regression. Since we have used the same cell lines in our *in vitro* and *in vivo* study, the diverse effects cannot be explained by genetic differences.

How can such a striking difference in drug response be explained? To start, tumor cells grown *in vitro* lack the complex microenvironment provided by neighboring cells, soluble secreted factors, and non-cellular matrix components [22]. Surrounding cells include tumor-, stromal-, immune- and endothelial cells, which mutually influence each other. Given that the constantly changing microenvironment controls many signaling pathways in tumor cells, it is not surprising that many *in vitro* observations do not correlate with *in vivo* observations [35]. For example, cells in culture divide once every day, while cells in primary human breast tumors divide less frequent (40–300 days) [36]. Importantly, the cell cycle is well controlled by many signaling pathways and therefore the heterogeneous microenvironment may potentially affect the action of taxanes on tumor cells. Indeed, immunohistological analysis and intravital imaging of mouse and human tumor tissues only revealed small increases in mitotic index following paclitaxel treatment [16]–[19], supporting our finding that mitotic perturbations are not causative for taxane-induced tumor regression [18], [19].

Interesting recent data suggest that the microenvironment contributes to drug responses via regulation of vascular permeability and innate immune cell infiltration [37]. It has indeed been hypothesized that immune cells might elicit an anti-tumor effect upon taxane administration [38] and macrophages can be directly activated by paclitaxel resulting in pro-inflammatory effects [39]. Although effects of the innate immune system cannot be excluded, the clear apoptotic effects of docetaxel administration in our immune-compromised and immune-proficient models indicate that the adaptive immune system is not the main contributor to taxane-dependent cell killing *in vivo*. However, to properly address this point, the effects of docetaxel-induced cell killing should be studied in both immune-deficient and immune-proficient mice originating from similar background strains.

In our study we observed that *in vivo* docetaxel treatment may in some cells affect mitotic progression and therefore induce apoptosis (Figure 4B), however, for the vast majority of cells, apoptosis is induced independently of its effect on mitosis (Figure 4C, S5C). The ability of docetaxel to kill tumor cells independent of its effects on mitosis could have important implications for the use and development of mitosis-specific drugs, such as Eg5 or Aurora A inhibitors (reviewed in [40]) and might explain the marginal anti-tumor effects of these drugs in patients until now [41], [42]. In addition, combination therapies designed *in vitro* to enhance mitotic defects induced by taxane treatment, might have to be reconsidered [43]. Nevertheless, tumor-specific traits other than an increased percentage of mitotic cells could still be exploited in future anti-cancer strategies. An example of such a trait is the presence of an abnormal chromosome number, called aneuploidy. More than 80% of all tumors harbor aneuploid cells and recently it was suggested that these cells could be specifically killed by enhancing proteotoxic stress [44].

Our findings illustrate that tumor cells in *in vitro* culture systems could respond differently to chemotherapy than their *in vivo* counterparts that are located in their natural environment. In addition, our data show how useful intravital imaging techniques are to study the true molecular mechanism of drugs that are frequently being used in the clinic. Therefore, future research in the specific cellular effects of taxanes and other (anti-mitotic) drugs would benefit from *in vivo* validation with techniques as presented in this study, which hopefully leads to the development of improved combination therapies that will enhance clinical efficacy.

## Materials and Methods

### Ethics statement

All mouse experiments were approved by the animal ethical committee (DEC) of the Netherlands Academy of Sciences (KNAW), the Netherlands, and animals were kept at the Hubrecht animal facility in Utrecht, the Netherlands. All surgeries and imaging sessions were performed under anesthesia using isoflurane and imaging sessions were limited to 3 hours per day to minimize suffering.

### Cell culture, cell lines and reagents

SW480 cells were obtained from the cryobank of the University Medical Center Utrecht and were previously described [43]. C26 cells were a kind gift of O. Kranenburg and were previously published [45]. Cells were grown in DMEM (Lonza), supplemented with 6% FCS (Clontech), pen/strep (Invitrogen) and ultraglutamine (Lonza). *In vitro* time lapse imaging of cells was performed in Leibovitz medium (Lonza) supplemented with 6% FCS, pen/strep and ultraglutamine. SW480 and C26 cell lines were infected with lentivirus carrying pLV.CMV.puro-c3-CAAX or pWPXLd.c3-CAAX respectively and pLV.CMV.puro.H2B-Dendra. Cell lines were selected with 2 µg/ml puromycin (after pLV.CMV.puro infection) and single colonies were selected after replating 1–2 cells/well or using FACS sorting. Puromycin and docetaxel (used in cell culture experiments) were from Sigma and zVAD-fmk (50 µM) was from Calbiochem.

### Plasmids

The caspase-3-CAAX FRET probe [23], [26] was ligated in pLV.CMV.puro or pWPXL.d. pLV.CMV.puro was linearized using Pst1 (+blunt with Klenow) and subsequently cut with EcoR1. pWPXL.d was linearized using BamH1 (+blunt) and subsequently cut with EcoR1. Caspase-3 CAAX was obtained after restriction of pcDNA3.c3-CAAX with HindIII (+Blunt) and EcoR1. H2B-Dendra2 was ligated in pLV.CMV.puro. Enzymes were all from NEB. Both pLV.CMV.puro and pWPXL.d were a kind gift from Dr. Patrick Derksen.

### Live cell imaging *in vitro*

Cells were plated in 8-well chambered glassbottom slides (LabTek) and imaged in a heated chamber at 37°C using either a Leica TCS SP5 AOBS two-photon microscope (Mannheim, Germany) or a DeltaVision RT system (Applied Precision) with a 20×/0.75NA objective (Olympus) using SoftWorx software. The stored images were analyzed using LasAF software or ImageJ. Unless indicated, docetaxel was added 10 minutes prior to filming. Fluorescent images were acquired every 30 minutes for a duration of 48 hours.

### DTX washout experiment

Cells were incubated with 1 µM docetaxel for a period of two hours, followed by three PBS-washing steps. After the addition of fresh medium, cells were imaged in a heated chamber (37°C and 5% CO2) using a DeltaVision RT system (Applied Precision) with a 20×/0.75NA objective (Olympus) using SoftWorx software. Image analysis was performed using ImageJ.

### Colony formation assay

Cells (∼50.000/well) were plated on 6-wells plates (Costar) (day 0). Docetaxel was added on day 1. On day 6, plates were washed with PBS, fixed for 5 minutes with 96% Methanol, stained with 0,1% crystal violet.dH20 and scanned for analysis. Quantification was performed using ImageJ image analysis.

### Window surgery

Window was placed 2–6 weeks after injection of 0,5 –1 million SW480 or C26 cells stably expressing both H2B-Dendra and the caspase-3 FRET probe. All surgical procedures were performed under 2% isoflurane inhalation anesthesia and under aseptic conditions. Before surgery, the tumor area was shaved and the skin was disinfected using 70% EtOH. An incision was made through the skin, where the imaging window was inserted (for details see [30], [46]). The window was secured with a non-absorbable, non-woven purse-string suture (4-0 prolene suture). After surgery the mice were kept at 32°C until fully recovered from anesthesia. Mice were closely monitored for a few hours after surgery and food was provided within the cage.

### Intravital imaging

Mice were sedated using isoflurane inhalation anesthesia (1.5% to 2% isoflurane/O_2_ mixture), and placed with their head in a facemask within a custom designed imaging box. The imaging box and microscope were kept at 32°C using a climate chamber surrounding the complete microscope stage, including the objectives. Mice were imaged for a maximum period of 3 hours per day. The mouse vitals were monitored during imaging using the MouseOx system (starr lifescience Corp, Oakmont, PA, USA). Imaging was performed on an inverted Leica TCS SP5 AOBS two-photon microscope (Mannheim, Germany) with a chameleon Ti:Sapphire pumped Optical Parametric Oscillator (Coherent Inc. Santa Clare, CA, USA). Docetaxel obtained from the University Medical Center Utrecht pharmacy was diluted in PBS to a stock concentration of 5 mg/ml. A single dose of 5 µl per gram mouse was administered intravenously to obtain a concentration of 25 mg/kg *in vivo*. PBS was used as vehicle control treatment and was administered similarly to docetaxel.

Image analysis was performed using ImageJ software. CFP-YFP ratios of single cells were determined by drawing specific regions of interest (ROIs) around cells and dividing the CFP average intensity by the YFP average intensity. Background levels for CFP and YFP were subtracted prior to ratio calculation.

### Caspase-3 CFP-YFP ratio imaging

CPF and YFP *in vitro* and *in vivo* images were acquired by only exciting CFP (*in vitro*: 405 nm, *in vivo*: 820 nm) as described above. Before every experiment, the PMT settings of very channel were optimized, but kept constant during the course of the multiple day experiment. The CFP-YFP ratio was analyzed using Leica TCS and ImageJ software. To compare different experiments and conditions, the relative CFP-YFP ratio was calculated by normalizing all ratios to the CFP-YFP ratio at the start of the experiment.

### FLIM analysis

FLIM analysis was carried out using a Leica TCS SP5 inverted microscope (Mannheim, Germany) with a 25× (HCX IRAPO N.A.0.95 WD 2.5 mm) water objective, which was adapted for TCSPC (time-correlated single-photon counting) FLIM with a Becker and Hickl SPC 830 card using 64 time channels. The samples were excited using a femtosecond titanium chameleon Ti:Sapphire pumped Optical Parametric Oscillator (Coherent Inc. Santa Clare, CA, USA (80 MHz). Images were obtained with a line-scan speed of 400 Hz. Two-photon excitation was carried out using a wavelength of 820 nm and fluorescence was detected between 450 nm and 480 nm. The fluorescence decays obtained were fitted using a single exponential decay model with Becker and Hickl SPCImage software v2,9,9, 29107, and the lifetimes were portrayed in false color maps.

### Isolation of tumors

Tissues were isolated and fixed in periodate-lysine-paraformaldehyde (PLP) buffer (2,5 ml 4% PFA+0,0212 g NaIO_4_+3,75 ml L-Lysine+3,75 ml P-buffer (pH 7.4)) O/N at 4 °C. On the following day, the fixed tissues were washed twice with P-buffer and placed for at least 6 hours in 30% sucrose at 4 °C. The tissues were then embedded in OCT tissue freezing medium (Jung) and stored at −20°C before sectioning.

### Phospho-Serine 10 Histone H3 staining (pHH3)

PLP tumor sections were pre-incubated with TBS-0,1% Tween20 4% BSA for 30 minutes at room temperature. Anti-phospho-Histone H3 (1∶250) was incubated for 3 hours in TBS-0,1% Tween20 4%BSA, washed twice with TBS-0,1% Tween20 and secondary antibody was incubated for 45 minutes in the presence of DAPI. Prolong Antifade was used to mount the slides and analysis was done on a DeltaVision RT system (Applied Precision) with 60× or 10× objective (Olympus) using SoftWorx software.

### Cleaved capase-3 staining

PLP tumor sections were rehydrated in PBS for 10 minutes at room temperature, after which tissue was blocked for 30 minutes in PBS with 5% NGS, 2,5% BSA, and 0,3% Triton X-100. Anti-cleaved-caspase-3 (1∶200) was incubated overnight in 0,5× blocking solution, washed three times with PBS and secondary antibody was incubated for 2 hours in 0,5× diluted blocking solution. After washing with PBS, tissues were mounted with Vectashield hard set mounting medium (H-1400, Vector Laboratories Inc., Burlingame, CA). Analysis was performed on a Leica TCS SP5 AOBS inverted microscope (Mannheim, Germany) with a 25× (HCX IRAPO N.A.0.95 WD 2.5 mm) water objective.

## Supporting Information

Figure S1 **Docetaxel control experiments.** A, body weight of vehicle (PBS) and docetaxel-treated mice, 1 day before intravenous injection of docetaxel or its PBS vehicle. The average shown is from 10 mice. B, representative images of apoptotic (defragmented) cells by intravital imaging, visualized by H2B Dendra2. Shown are vehicle (PBS) (left) and docetaxel-treated (right) tumors before and after treatment. A few apoptotic (defragmented) cells are indicated with red circles. C, quantification of the number of apoptotic cells before or 2 days after a single intravenous injection of vehicle (PBS). The docetaxel-treated animals are shown in Figure 1B. Average of 3 independent experiments + SEM is indicated.
(TIF)

Figure S2 **The caspase-3 FRET sensor reflects caspase-3 activation, and is not influenced by dendra2 photomarking or cell cycle progression.** A, quantification of CFP-YFP ratio before and after H2B-D switching *in vitro*. The n = 10 cells per condition + SEM. B, top: Representative stills of a SW480 cell stably expressing H2B-D (red) and caspase-3 FRET sensor (green) progressing through interphase and mitosis without addition of drugs. Time in hours before mitosis is shown. T = 0.00 indicates mitotic entry. Bottom: quantification of CFP-YFP ratio of cells progressing through the cell cycle. n = 11 cells. Average + SD is shown. C, the graph represents the CFP-YFP ratio of cells that are positively and negatively stained for cleaved caspase-3 (activated form). All values are normalized to the average CFP-YFP ratio of cells negative for cleaved caspase-3. The n = 30 cells (randomly picked from 5 positions), each dot represents 1 cell. Line indicates average + SEM. ***: significant (student t test, unpaired, p<0.001).
(TIF)

Figure S3 **Vehicle (PBS) treatment does not induce caspase-3 activity.** Tumor cells expressing the caspase-3 FRET biosensor were imaged intravitally by exciting CFP and detecting CFP and YFP or CFP lifetime (see Materials and Methods). A, the graph in which the CFP-YFP ratios (normalized to average CFP-YFP value before treatment) of individual cells *in vivo* are plotted at indicated times after a single intravenous injection of vehicle (PBS). For the docetaxel treatment, see Figure 3B. The different symbols represent different individual mice and every symbol represents one cell. The n = 3 mice. Line indicates median + IQR. ***: significant (Mann Whitney U test, p<0.008). B, FLIM analysis of single cells (dots) in sections of indicated parts of the tumor. Tumor was isolated two days after docetaxel treatment. ‘Inside’ indicates outer part of tumor (opposite of the imaging window site). ‘Middle’ indicates middle part of tumor (cross section). ‘Window’ indicates part of tumor on which the imaging window was placed. Each dot represents one cell. Line indicates median + IQR. C, the CFP fluorescent lifetime of vehicle (PBS)-treated C26 cells *in vivo* plotted at indicated time points. One dot represents one cell. ***: significant (Mann Whitney U test, p<0.0001). Line indicates median + IQR.
(TIF)

Figure S4 **Caspase-3 inhibition abolishes docetaxel-induced increase in the number of cells with apoptotic CFP-YFP ratio** ***in vitro*** **.** A, representative stills of mitotic SW480 cells *in vitro* in the absence (top row) or presence of 1 µM (middle row) or 2 nM (bottom row) docetaxel, resulting in a mitotic delay (middle) or abnormal mitotic exit (bottom). Time after onset of mitosis is shown in each panel (hr:min). Black arrows indicate (abnormal) mitotic exit, yellow arrows indicate abnormal nuclei. B, quantification of the CFP-YFP ratio of SW480 cells during mitotic delay *in vitro* following the addition of DMSO (left) or pan-caspase inhibitor zVAD-fmk (50 µM) in the presence of 1 µM docetaxel. The graph shows the CFP-YFP ratios of treated SW480 cells over time. Cells with a CFP-YFP ratio above the apoptotic CFP-YFP ratio (>1.3 times increase) are indicated with gray lines, all other cells with black lines. The n>10 cells per graph. Arrows indicate the onset of mitosis. C, quantification of the CFP-YFP ratio of interphase SW480 cells in cell culture in the absence (blue lines) or presence (red lines) of 1 µM docetaxel.
(TIF)

Figure S5 **Docetaxel treatment** ***in vivo*** **does not cause an increase in mitotic cells, but does affect chromosome alignment.** A, immunohistochemical images of C26 tumor slides stained with a Phospho-Serine 10 Histone H3 (pHH3) antibody to visualize mitotic figures. Individual channels of DAPI (blue), pHH3 (red) and merge images are shown of a representative field. Scale bars represent 20 µm. B, left: Images of slides from vehicle (PBS)-treated (top) or docetaxel-treated (bottom) tumors stained for pHH3 two days after treatment *in vivo*. Scale bars represent 50 µm. Right: Quantification of the percentage of pHH3-positive cells in vehicle (PBS)-treated and docetaxel-treated tumors, n indicates number of cells. C, left: Representative images of aligned, mild misaligned and severe misaligned chromosomes. Dotted lines indicate the alignment of the chromosomes. Scale bars represent 3 µm. Right: Quantification of chromosome alignment (normal alignment in black, mild misalignment in gray, severe misalignment in red) in vehicle (PBS)-treated and docetaxel-treated tumors, n indicates number of cells.
(TIF)

Figure S6 **Short-term exposure to docetaxel causes mitotic delay** ***in vitro*** **.** A, representative *in vivo* images of H2B-D-expressing SW480 and C26 cells as used for the assessment of nuclear morphology in Figure 5B. B, the percentage of SW480 cells with an apoptotic CFP-YFP ratio plotted at indicated time points after single intravenous administration of the vehicle (PBS). The n indicates number of cells analyzed. C, graphs are shown in which the duration of mitosis in C26 and SW480 cells is plotted against indicated conditions. Cells were incubated with or without 1 µM docetaxel for 2 hours followed by 3 PBS-washing steps. One dot represents one cell. ***: significant (Mann Whitney U test, p<0.0001). Line indicates median + IQR. The n indicates number of cells analyzed.
(TIF)
