# Supplementary figures and images for: Intravital FRET Imaging of Tumor Cell Viability and Mitosis during Chemotherapy
Source: PLoS One. 2013 May 15;8(5):e64029. doi: 10.1371/journal.pone.0064029 (PMC3654962; doi:10.1371/journal.pone.0064029)

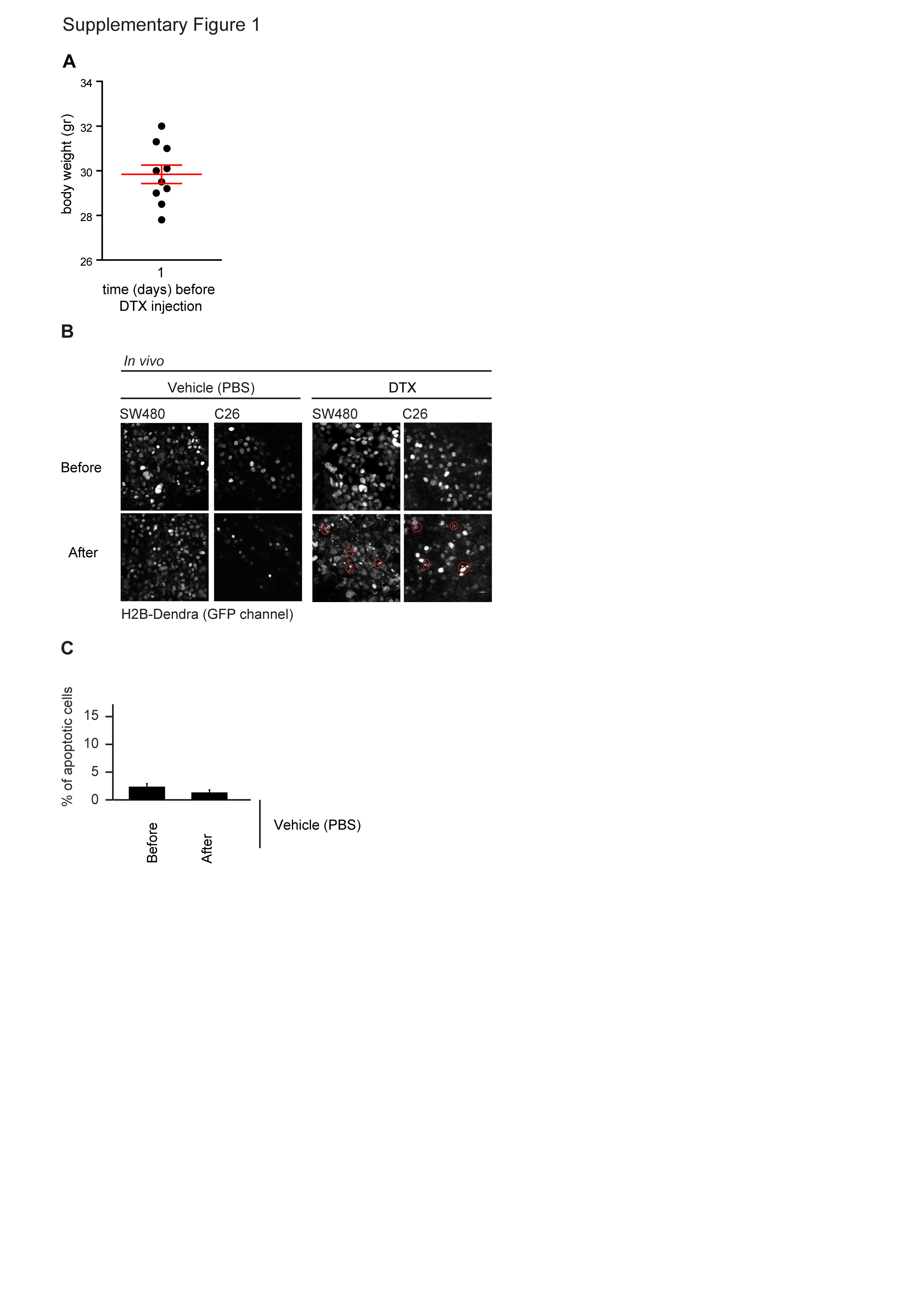

Supplement: Figure S1 — Docetaxel control experiments. A, body weight of vehicle (PBS) and docetaxel-treated mice, 1 day before intravenous injection of docetaxel or its PBS vehicle. The average shown is from 10 mice. B, representative images of apoptotic (defragmented) cells by intravital imaging, visualized by H2B Dendra2. Shown are vehicle (PBS) (left) and docetaxel-treated (right) tumors before and after treatment. A few apoptotic (defragmented) cells are indicated with red circles. C, quantification of the number of apoptotic cells before or 2 days after a single intravenous injection of vehicle (PBS). The docetaxel-treated animals are shown in Figure 1B. Average of 3 independent experiments + SEM is indicated. (TIF) [file pone.0064029.s001.tif]

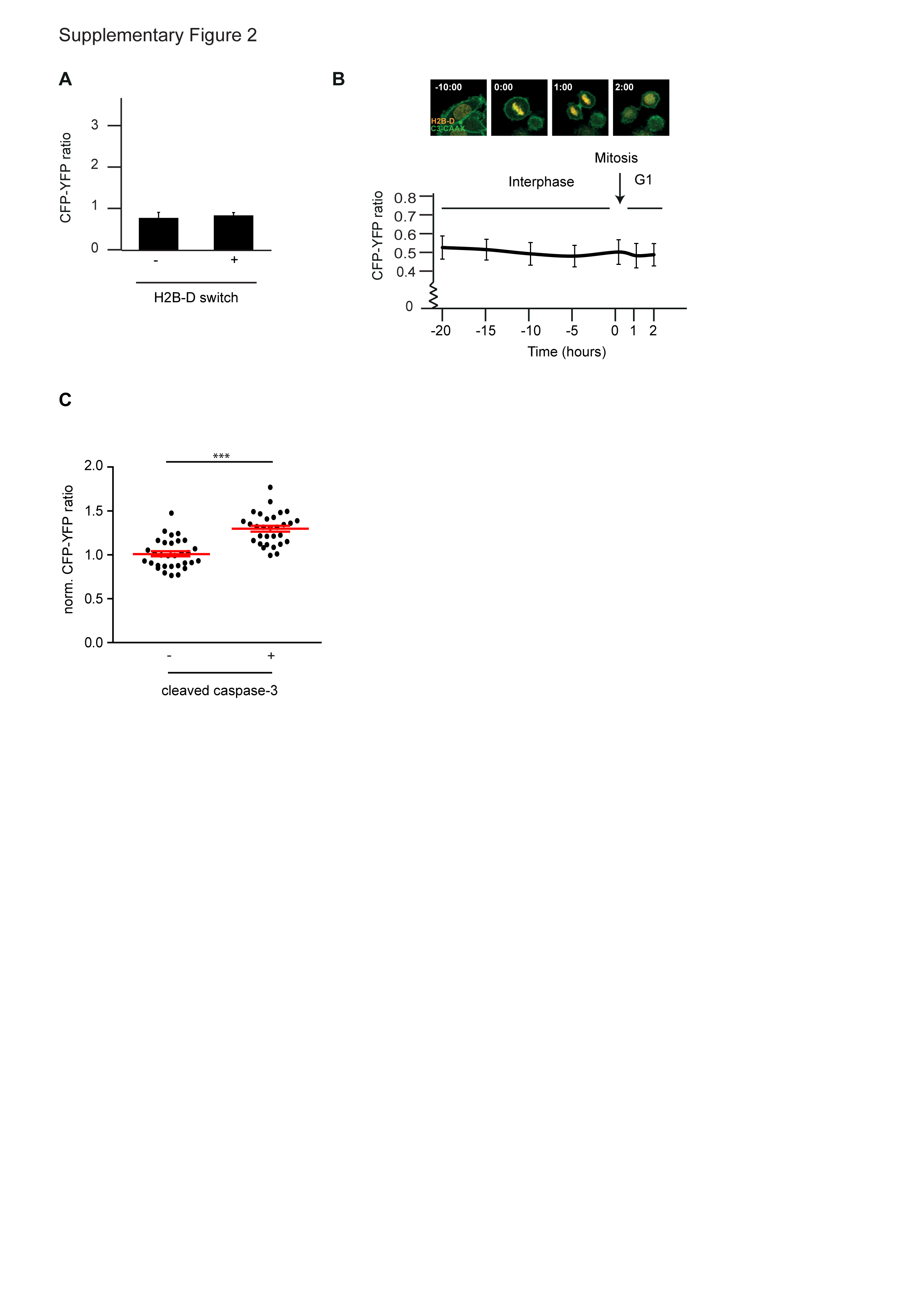

Supplement: Figure S2 — The caspase-3 FRET sensor reflects caspase-3 activation, and is not influenced by dendra2 photomarking or cell cycle progression. A, quantification of CFP-YFP ratio before and after H2B-D switching in vitro. The n = 10 cells per condition + SEM. B, top: Representative stills of a SW480 cell stably expressing H2B-D (red) and caspase-3 FRET sensor (green) progressing through interphase and mitosis without addition of drugs. Time in hours before mitosis is shown. T = 0.00 indicates mitotic entry. Bottom: quantification of CFP-YFP ratio of cells progressing through the cell cycle. n = 11 cells. Average + SD is shown. C, the graph represents the CFP-YFP ratio of cells that are positively and negatively stained for cleaved caspase-3 (activated form). All values are normalized to the average CFP-YFP ratio of cells negative for cleaved caspase-3. The n = 30 cells (randomly picked from 5 positions), each dot represents 1 cell. Line indicates average + SEM. ***: significant (student t test, unpaired, p<0.001). (TIF) [file pone.0064029.s002.tif]

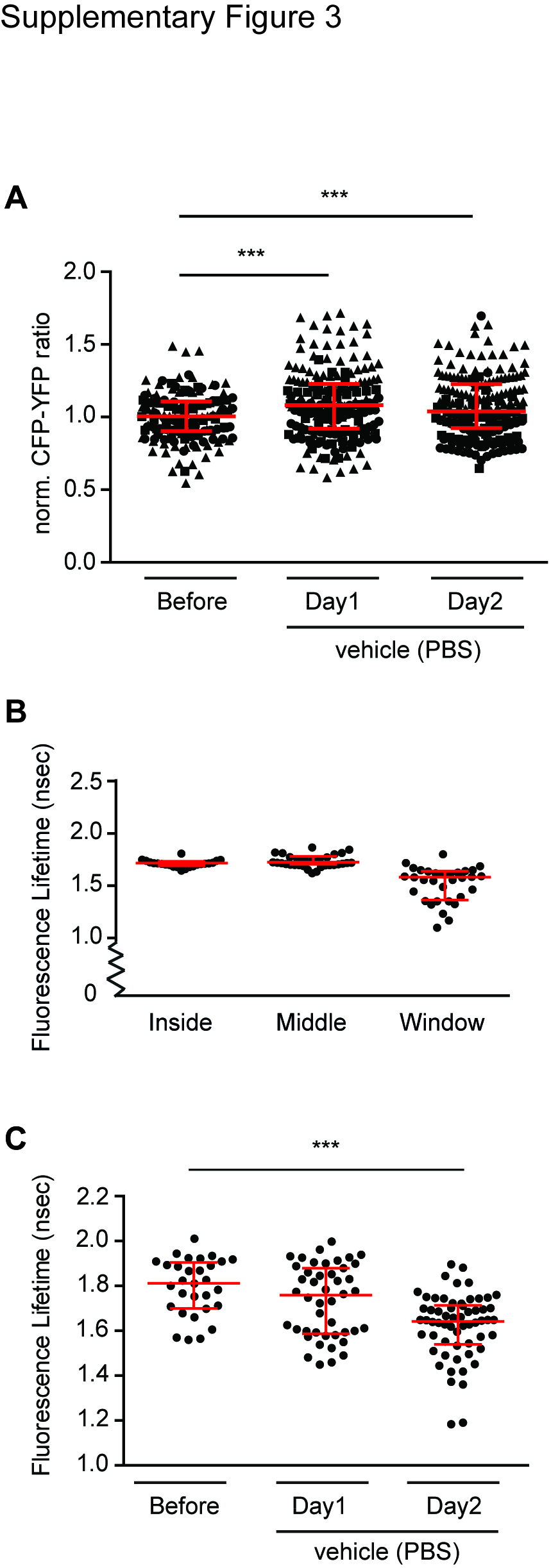

Supplement: Figure S3 — Vehicle (PBS) treatment does not induce caspase-3 activity. Tumor cells expressing the caspase-3 FRET biosensor were imaged intravitally by exciting CFP and detecting CFP and YFP or CFP lifetime (see Materials and Methods). A, the graph in which the CFP-YFP ratios (normalized to average CFP-YFP value before treatment) of individual cells in vivo are plotted at indicated times after a single intravenous injection of vehicle (PBS). For the docetaxel treatment, see Figure 3B. The different symbols represent different individual mice and every symbol represents one cell. The n = 3 mice. Line indicates median + IQR. ***: significant (Mann Whitney U test, p<0.008). B, FLIM analysis of single cells (dots) in sections of indicated parts of the tumor. Tumor was isolated two days after docetaxel treatment. ‘Inside’ indicates outer part of tumor (opposite of the imaging window site). ‘Middle’ indicates middle part of tumor (cross section). ‘Window’ indicates part of tumor on which the imaging window was placed. Each dot represents one cell. Line indicates median + IQR. C, the CFP fluorescent lifetime of vehicle (PBS)-treated C26 cells in vivo plotted at indicated time points. One dot represents one cell. ***: significant (Mann Whitney U test, p<0.0001). Line indicates median + IQR. (TIF) [file pone.0064029.s003.tif]

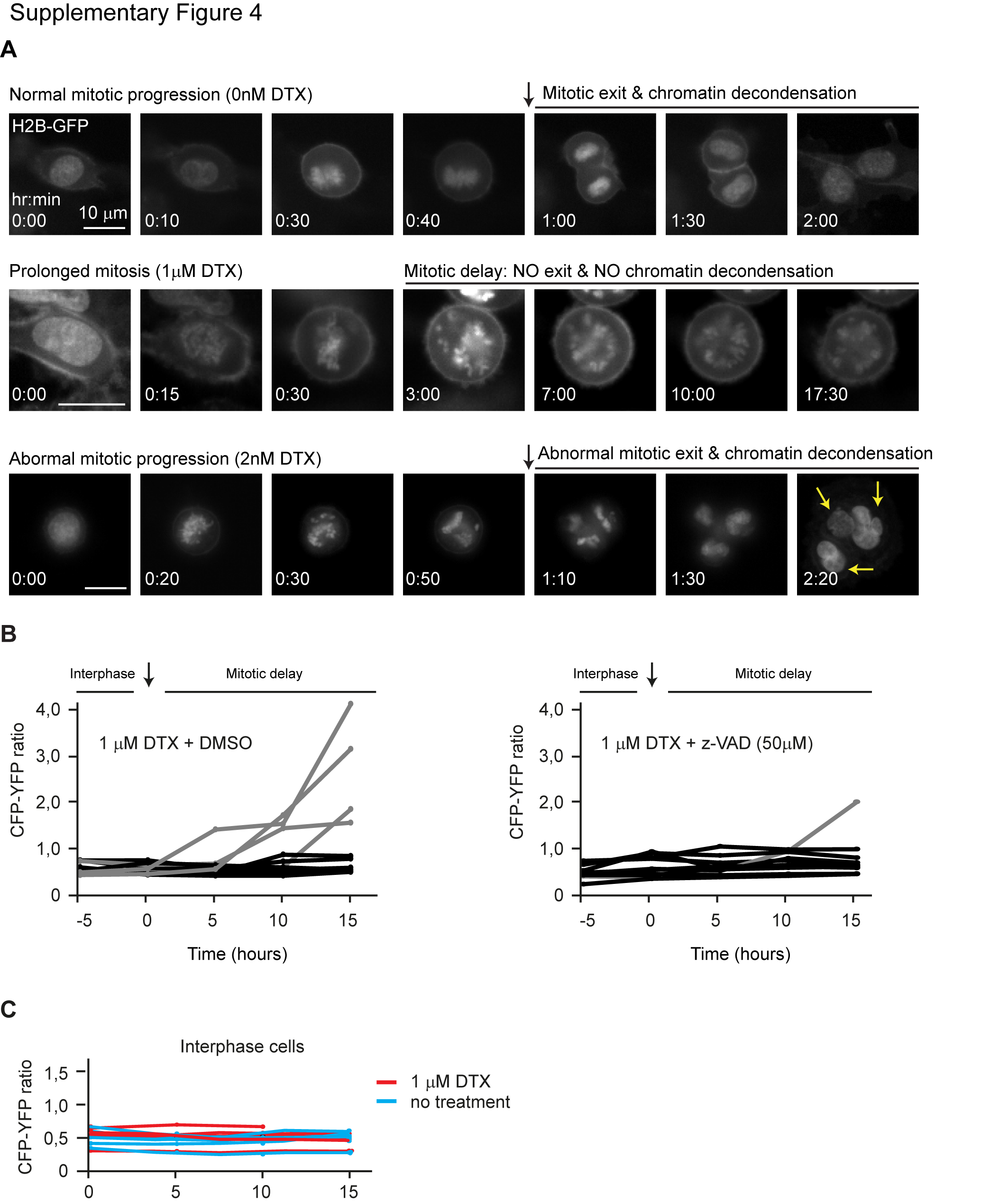

Supplement: Figure S4 — Caspase-3 inhibition abolishes docetaxel-induced increase in the number of cells with apoptotic CFP-YFP ratio in vitro . A, representative stills of mitotic SW480 cells in vitro in the absence (top row) or presence of 1 µM (middle row) or 2 nM (bottom row) docetaxel, resulting in a mitotic delay (middle) or abnormal mitotic exit (bottom). Time after onset of mitosis is shown in each panel (hr:min). Black arrows indicate (abnormal) mitotic exit, yellow arrows indicate abnormal nuclei. B, quantification of the CFP-YFP ratio of SW480 cells during mitotic delay in vitro following the addition of DMSO (left) or pan-caspase inhibitor zVAD-fmk (50 µM) in the presence of 1 µM docetaxel. The graph shows the CFP-YFP ratios of treated SW480 cells over time. Cells with a CFP-YFP ratio above the apoptotic CFP-YFP ratio (>1.3 times increase) are indicated with gray lines, all other cells with black lines. The n>10 cells per graph. Arrows indicate the onset of mitosis. C, quantification of the CFP-YFP ratio of interphase SW480 cells in cell culture in the absence (blue lines) or presence (red lines) of 1 µM docetaxel. (TIF) [file pone.0064029.s004.tif]

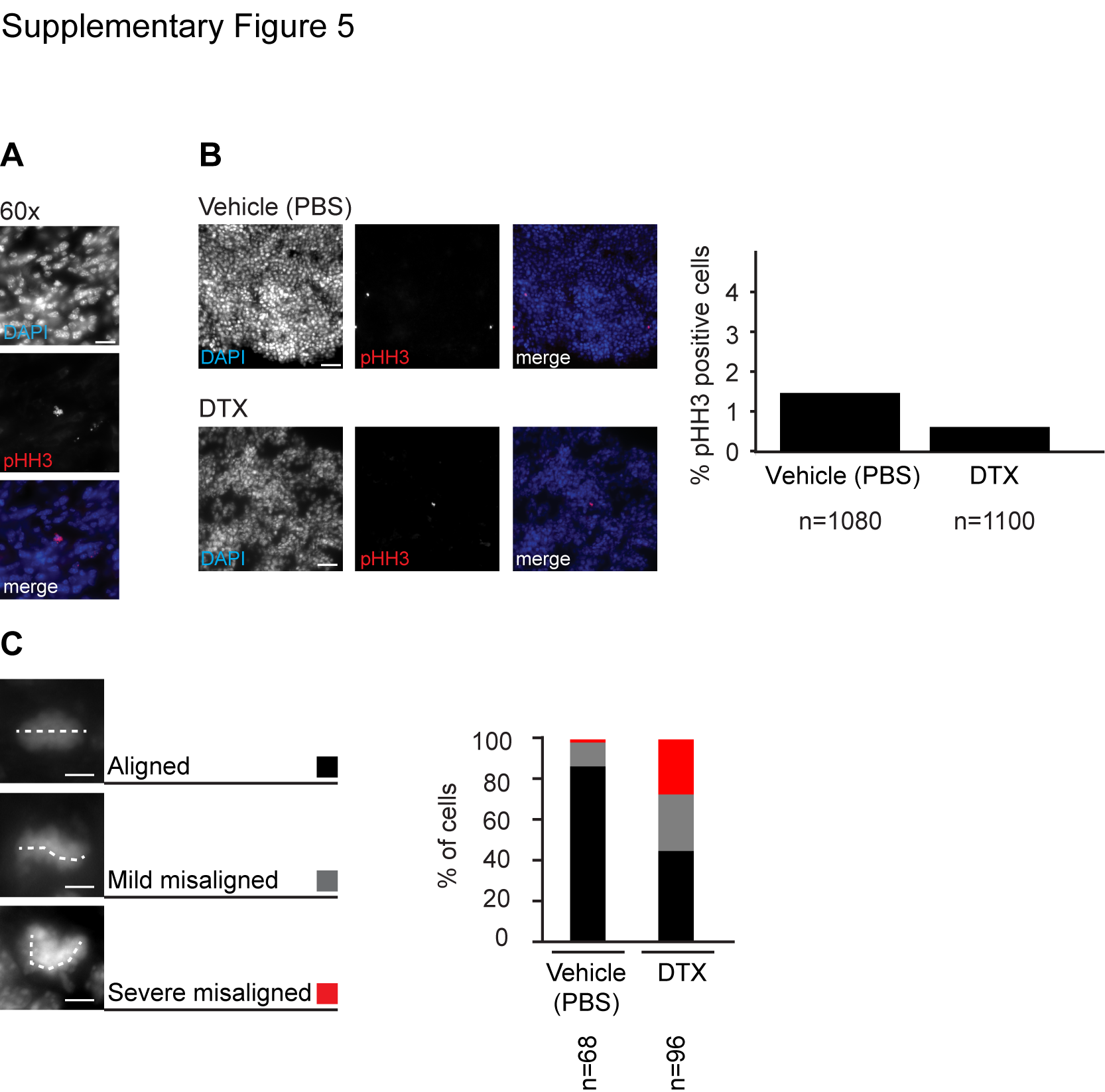

Supplement: Figure S5 — Docetaxel treatment in vivo does not cause an increase in mitotic cells, but does affect chromosome alignment. A, immunohistochemical images of C26 tumor slides stained with a Phospho-Serine 10 Histone H3 (pHH3) antibody to visualize mitotic figures. Individual channels of DAPI (blue), pHH3 (red) and merge images are shown of a representative field. Scale bars represent 20 µm. B, left: Images of slides from vehicle (PBS)-treated (top) or docetaxel-treated (bottom) tumors stained for pHH3 two days after treatment in vivo. Scale bars represent 50 µm. Right: Quantification of the percentage of pHH3-positive cells in vehicle (PBS)-treated and docetaxel-treated tumors, n indicates number of cells. C, left: Representative images of aligned, mild misaligned and severe misaligned chromosomes. Dotted lines indicate the alignment of the chromosomes. Scale bars represent 3 µm. Right: Quantification of chromosome alignment (normal alignment in black, mild misalignment in gray, severe misalignment in red) in vehicle (PBS)-treated and docetaxel-treated tumors, n indicates number of cells. (TIF) [file pone.0064029.s005.tif]

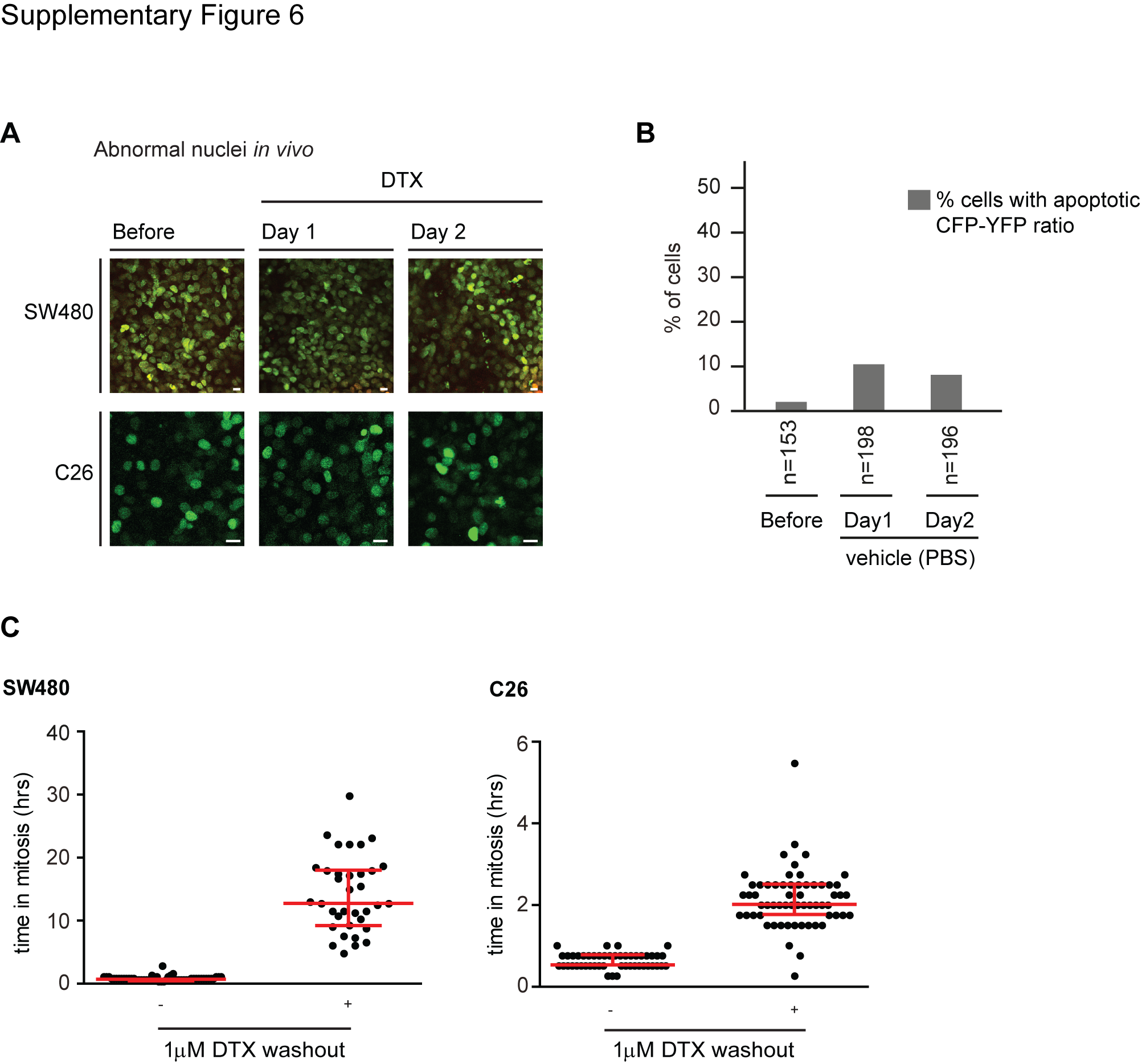

Supplement: Figure S6 — Short-term exposure to docetaxel causes mitotic delay in vitro . A, representative in vivo images of H2B-D-expressing SW480 and C26 cells as used for the assessment of nuclear morphology in Figure 5B. B, the percentage of SW480 cells with an apoptotic CFP-YFP ratio plotted at indicated time points after single intravenous administration of the vehicle (PBS). The n indicates number of cells analyzed. C, graphs are shown in which the duration of mitosis in C26 and SW480 cells is plotted against indicated conditions. Cells were incubated with or without 1 µM docetaxel for 2 hours followed by 3 PBS-washing steps. One dot represents one cell. ***: significant (Mann Whitney U test, p<0.0001). Line indicates median + IQR. The n indicates number of cells analyzed. (TIF) [file pone.0064029.s006.tif]
